# Endoplasmic reticulum stress induced by an ethanol extract of Coicis semen in Chang liver cells

**DOI:** 10.1186/s12906-018-2175-z

**Published:** 2018-03-20

**Authors:** Hwa Yeon Kim, Ha Na Song, Munkhtugs Davaatseren, Hyun Joo Chang, Hyang Sook Chun

**Affiliations:** 1 Advanced Food Safety Research Group, BK21 Plus, School of Food Science and Technology, Chung-Ang University, 4726 beon-gil, Seodong-daero, Daedeok-myeon, Anseong-si, 17546 Gyeonggi-do Republic of Korea; 2 Research Group of Food Safety, Korea Food Research Institute, 62, 1201 beon-gil, Anyangpangyo-ro, Bundang-gu, Seongnam-si, Gyeonggi-do Republic of Korea

**Keywords:** Metabolic disease, Endoplasmic reticulum stress, Coicis semen, Coixol

## Abstract

**Background:**

It is well known that endoplasmic reticulum (ER) stress plays a huge role in development of metabolic diseases. Specially, ER stress-induced cellular dysfunction has a significant involvement in the pathogenesis of human chronic disorders.

This study was designed to study to assess whether an ethanol extract of Coicis Semen (CSE) and coixol induces the ER stress in Chang liver cells.

**Methods:**

Coicis Semen was mixed with 95% ethanol at a ratio of 1:10 (*w*/*v*) and freeze dried. Chang liver cells were seeded to 96-well plates and treated with or without CSE (100, 200, 300, 500, or 1000 μg/mL) or coixol (100, 200, 300, 500, 750, or 1000 μg/mL). cell viability was analyzed with MTT assay. Effects of CSE and coixol on expression of the genes for ER stress markers were determined with qRT-PCR and the expression of the protein levels of ER stress markers were determined with western blotting.

**Results:**

The concentration causing 50% inhibition (IC_50_) for CSE and coixol was 250 and 350 μg/mL, respectively. The CSE and coixol increased the gene expression of BiP and CHOP in a dose-dependent manner. Furthermore, CSE and coixol dose-dependently increased the the expression of XBP1.

**Conclusions:**

CSE or coixol may have cytotoxic effect to Chang liver cells and, may induce ER stress and stimulate the UPR via activation of the PERK and IRE1 pathways in normal liver cells.

**Electronic supplementary material:**

The online version of this article (10.1186/s12906-018-2175-z) contains supplementary material, which is available to authorized users.

## Background

There has been growing interest in the study of chronic metabolic syndrome, which has become one of the hot topic in the world. Particularly, effort to elucidate the mechanism of human metabolic syndrome at the cellular level have demonstrated an association between endoplasmic reticulum (ER) stress and certain metabolic diseases, which revealed that ER stress-mediated cell dysfunction is involved in the pathogenesis of human chronic disorders, including type 2 diabetes, atherosclerosis, and neurodegeneration [1]. According to So et al., [2], increased expression of the unfolded protein response (UPR)-regulated genes, such as binding immunoglobulin protein (BiP) and X-box binding protein 1 (XBP1), is associated with several types of cancer, and presumably enhance the metastatic capability and growth ability of cancer cells. Given this relationship between ER stress and various diseases, ER stress has attracted attention as an important key factor for assessing toxicity at the cellular level.

The ER is a major organelle in eukaryotic cells, which involves with synthesis, folding, and transfer of proteins, post-translational modification, and control of intracellular Ca^2+^ concentration. In an abnormal environment, ER can induce an adaptive response, referred to as the UPR, to relieve environmentally induced stress. The UPR is roughly classified into four phases: inhibition of protein synthesis, increased protein-folding capacity, removal of unfolded proteins by proteasomes (ER-associated degradation, ERAD), and initiation of apoptosis [3]. ER stress triggers the UPR pathway to activate inositol-requiring protein 1 (IRE1), protein kinase-like endoplasmic reticulum kinase (PERK), and activating transcription factor 6 alpha (ATF6α). Phospho-IRE1 splices XBP1 mRNA, which acts as a transcription factor to promote the gene expression of ER stress-related molecules such as chaperones in the nucleus. Phospho-PERK (p-PERK) induces phosphorylation of eukaryotic translation initiation factor 2 alpha (p-eIF2α), and activated eIF2α inhibits the synthesis of protein [4]. The active form of ATF6α moves to the Golgi complex, where it is cleaved by SP1 and two proteins in the Golgi membrane [5]. Cleaved ATF6α is transferred to the nucleus and increases the expression of ER stress markers such as BiP and CCAAT/enhancer binding protein homologous protein (CHOP).

Coicis Semen is widely consumed as an herbal medicine and nourishing food in China and South Korea. It is obtained by removing the peel and drying the seeds of *Coixlacryma-jobi* Linne var. *mayuen* Stapf of family Gramineae. Coicis Semen has been reported to have a variety of functions, including inhibition of diabetic nephropathy [6], an anti-hyperlipidemic effect [7] in rats, anti-inflammatory effects in lipopolysaccharide-stimulated Raw 264.7 cells [8], and anti-cancer effects in human lung cancer cells [9, 10]. Coicis Semen contains a variety of functional chemicals and can be used as food or food components.

Generally, it believed that herbal medicines or herb extracts are safe for consumption, it is not recommended to use for long term without sufficient scientific studies. A recent study reported that the side effects of traditional medicine and herbal extracts were used as functional food [11]. For this reason, the US Food and Drug Administration found that *Ephedra* causes adverse side effects, including headache, hypertension, and insomnia, which resulted ban of *Ephedra* in 2004, which had been used as a dietary supplement. Correspondingly, aristolochic acid found in *Aristolochiae Fangchi Radix* causes nephropathy [12, 13] and has been reported as a potent human carcinogen [14]. Similarly, there have been several reports on embryotoxicity of Coicis Semen and its detrimental effects [15, 16]. According to Tzeng et al., [16], a water extract of Coicis Semen may induce embryotoxicity in pregnant rats. However, only a few studies have examined whether Coicis Semen has toxic effects. Thus, studies are needed to investigate whether Coicis Semen has toxic effects via elucidating the potential toxicity resulted from ER stress and its UPR-mediated genes expressions.

The aim of the present study was to investigate whether an ethanol extract of Coicis Semen (CSE) and coixol induces the ER stress in Chang liver cells. To examine the potential cytotoxic and effects of Coicis Semen on the ER, we performed quantitative reverse transcription polymerase chain reaction (qRT-PCR) and western blot analysis.

## Methods

### Materials

Coixol (6-methoxy-1,3-benzoxazol-2(3H)-one), tunicamycin, sodium 4-phenylbutyrate (4-PBA), and 3-(4,5-dimethylthiazol-2-yl)-2,5-diphenyltetrazolium bromide (MTT) were obtained from Sigma Aldrich (St. Louis, MO, USA). Primary antibodies against ATF6α, phospho-PERK, and β-actin, and horseradish peroxidase (HRP)-conjugated secondary antibodies against rabbit and mouse immunoglobulins, were purchased from Santa Cruz Biotechnology Inc. (Dallas, TX, USA). Primary antibodies against phospho-eIF2α, CHOP, and BiP were purchased from Cell Signaling Technology Inc. (Danvers, MA, USA). Enhanced chemiluminescent reagent and buffers, such as TBST and gel-forming buffer used in western blot analysis, were from Bio-Rad (Hercules, CA, USA). Culture media and other cell culture related materials were obtained from Gibco (Grand lsland, NY, USA).

### Preparation of an ethanol extract of Coicis semen (CSE)

Coicis Semen was purchased from a local market in South Korea, authenticated (Ho Cheol Kim, Kyung Hee University) and stored at Chung-Ang University Food Safety Laboratory (voucher CAUNP10). Coicis Semen extract was prepared as described in our previous study with minor modifications [17]. Briefly, Coicis Semen (2 kg) was mixed with 95% ethanol (20 L) at a ratio of 1:10 (*w*/*v*). The mixture was stirred at room temperature for 3 h and then was filtered using a filter paper. The filtrate was evaporated under reduced pressure to remove the ethanol and then lyophilized for 3 days using a freeze dryer (2.6% yield). The freeze dried sample was stored at − 20 °C.

### High-performance liquid chromatography (HPLC) **analysis**

CSE solutions (0.913 mg/mL) and coixol standards (10, 50, and 100 μg/mL) were dissolved in the mobile phase comprising 0.1% phosphoric acid and acetonitrile (75:25, *v*/v). An Agilent 1200 Infinity Series instrument (Agilent Technologies, CA, USA) equipped with a UV detector was used for HPLC. A Phenomenex SynergiHydro-RP column (4.6 × 250 mm, Torrance, CA, USA) was used for separation. The HPLC conditions were as follows: injection volume, 10 μL; column temperature, 25 °C; flow rate, 1 mL/min; UV detection, 232 nm. The concentration of coixol in the extract was calculated upon standard curves plotted according to the linear regression analysis of the integrated peak areas versus concentrations for the coixol.

### Cell culture condition and cell viability assay

Chang liver cells were provided by Seoul National University (Seoul, Korea). The culture medium was Dulbecco’s modified Eagle’s medium containing 10% heat-inactivated fetal bovine serum (FBS) and 1% 100 U/mL penicillin− 100 μg/mL streptomycin. Cells were incubated at 37 °C under 5% CO_2_. Chang liver cells were seeded to a concentration of 1 × 10^5^ cells/mL in 96-well plates and incubated for 24 h. After incubation, CSE (100, 200, 300, 500, or 1000 μg/mL) or coixol (100, 200, 300, 500, 750, or 1000 μg/mL) dissolved in 0.5% dimethyl sulfoxide (DMSO) was added to the cells in serum-free medium, and the cells were incubated for 48 h at 37 °C. Four hours before the end of the treatment, 20 μL of 5 mg/mL MTT solution (in PBS) was added to each well, and the cells were incubated for 4 h in the dark. Absorbance was measured at 595 nm using a Thermomax microplate reader (Molecular Devices, Sunnyvale, CA, USA). Cell viability was analyzed using Origin Pro 8.0, and the data are expressed relative to the vehicle control, which was set at 100%.

### qRT-PCR conditions and primer sequences

Chang liver cells (2 × 10^5^ cells/well) were seeded in 6-well plates for 24 h. The cells were treated with CSE (200, 300, 400, or 500 μg/mL) or coixol (100, 200, or 300 μg/mL) for 12 h and RNA was extracted from the harvested cells using an RNeasy kit (Qiagen, Hilden, Germany). RNA was reverse-transcribed to synthesize cDNA using a QuantiTect Reverse Transcription kit (Qiagen). The experiment was conducted using a CFX96 Real-Time PCR Detection system (Bio-Rad). The PCR conditions were as follows: 40 cycles of predenaturation at 95 °C for 3 min; denaturation at 95 °C for 15 s; annealing at 60 °C for 10 s; and extension at 72 °C for 30 s. The expression of genes for ER stress markers was examined using Bio-Rad CFX Manager (Bio-Rad) and β-actin was used as the reference gene for normalization. The data are expressed relative to the expression of the vehicle control (0.5% DMSO).

The following primers were used: BiP, 5’-GGTGACCTGGTACTGCTTGATG-3′ (sense), 5’-CCTTGGATTCAGTTTGGTCATG-3′ (antisense); CHOP, 5’-CTTGGCTGACTGAGGAGGAG-3′ (sense), 5’-TCACCATTCGGTCAATCAGA-3′ (antisense); XBP1, 5’-CCTTGGATTCAG TTTGGTCATG-3′ (sense), 5-'GGGGCTTGGTATATATGTGG-3′ (antisense); unspliced XBP1, 5’-CAGCACTCAGACTACGTGCA-3′ (sense), 5’-ATCCATGGGGAGATGTTCTGG-3′ (antisense); spliced XBP1, 5’-CTGAGTCCGAATCAGGTGCAG-3′ (sense), 5’-ATCCATGGGGAGATGTTCTGG-3′ (antisense); EDEM, 5’-CAAGTGTGGGTACGCCACG-3′ (sense), 5’-AAAGAAGCTCTCCATCCGGTC-3′ (antisense); and β-actin, 5’-TCATCACCATTGGCAATGAG-3′ (sense), 5’-CACTGTGTTGGCGTACAGGT-3′ (antisense).

### Splicing of XBP1 mRNA cut by PstI

Amplified XBP1 mRNA was mixed with the restriction enzyme *Pst*I in reaction buffer and water, and the mixture was incubated at 37 °C for 2 h. The restriction site of *Pst*I is an intron of XBP1 mRNA. After cooling on ice for 5 min, the mixture was heated at 80 °C for 20 min to inactivate *Pst*I. The reaction product was loaded onto a 4% agarose gel inLoading STAR solution (Dynebio, Seongnam, Korea), and electrophoresis was conducted at 100 V for 1 h. The DNA fragment pattern was analyzed using a Gel Doc EZ imager (Bio-Rad).

### Western blot analysis

Chang cells were seeded at 1 × 10^6^ cells/well in a 100-mm culture dish and incubated for 24 h. The incubated cells were treated with 100, 200, or 300 μg/mL of coixol or CSE with or without 1 mM of 4-PBA for 24 or 48 h. The cells were harvested in phosphate buffered saline (PBS) containing 0.1% protease inhibitor cocktail (Quartett, Berlin, Germany), and the supernatant was removed. The cell pellet was mixed with radioimmunoprecipitation assay (RIPA) buffer containing 1% protease inhibitor cocktail. After mixing and cooling, the sample was centrifuged at 12,000 rpm for 20 min to extract protein. The protein was loaded onto a 10% or 12% polyacrylamide gel and electrophoresis was performed. The separated proteins were transferred to a nitrocellulose membrane using a SE22 transfer tank (Hoefer, MA, USA). We used primary antibodies targeting BiP, CHOP, and p-eIF2α (Cell Signaling Technology), and p-PERK, ATF6α and β-actin (Santa Cruz Biotechnology). The expression of protein was measured from X-ray film (GE Healthcare, Little Chalfont, UK) using Smart Chemi 500 (Sage Creation, Beijing, China) and quantified using Lane ID (ver. 4.0).

### Statistical analysis

All experiments were performed at least three times independently. The data are presented as the mean ± standard error of the mean (SEM) except for the cell proliferation results, which are presented as the mean ± standard deviation (SD). Statistical analysis was carried out using one-way analysis of variance (ANOVA) followed by Duncan’s multiple comparison test and the *p* value < 0.05 was considered as significant.

## Results

### Cytotoxicity of CSE and coixol in Chang liver cells

**Fig. 1:**
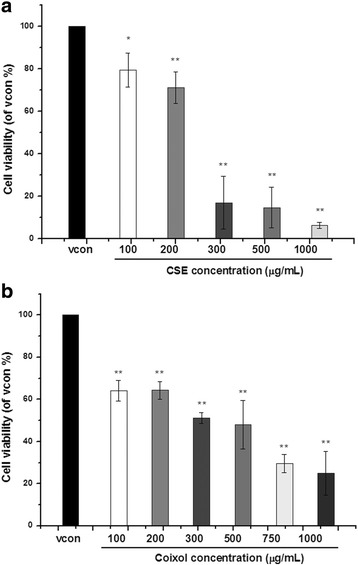
Inhibition of the proliferation of Chang liver cells by an ethanol extract of Coicis Semen (CSE) and coixol. The IC_50_ values for CSE and coixol were calculated as 250 and 350 μg/mL, respectively. (**a**) CSE (100, 200, 300, 500, or 1000 μg/mL) and (**b**) Coixol (100, 200, 300, 500, 750, or 1000 μg/mL), respectively. *: *p* < 0.05, **: *p* < 0.01, compared with the control group

The MTT assay was used to estimate the cytotoxicity of CSE and coixol in Chang liver cells. As shown in Fig. 1, CSE decreased cell viability in a dose-dependent manner compared with the vehicle control. Coixol tended to increase cytotoxicity with increasing concentration, but there was no clear difference among coixol concentrations tested. The concentration causing 50% inhibition (IC_50_) for CSE and coixol was determined as 250 and 350 μg/mL, respectively. The content of coixol in the CSE used in our study was estimated to be 30.1 μg/g by HPLC analysis (Additional file 1: Figure S1).

### Effects of CSE and coixol on expression of the genes for ER stress markers

**Fig. 2:**
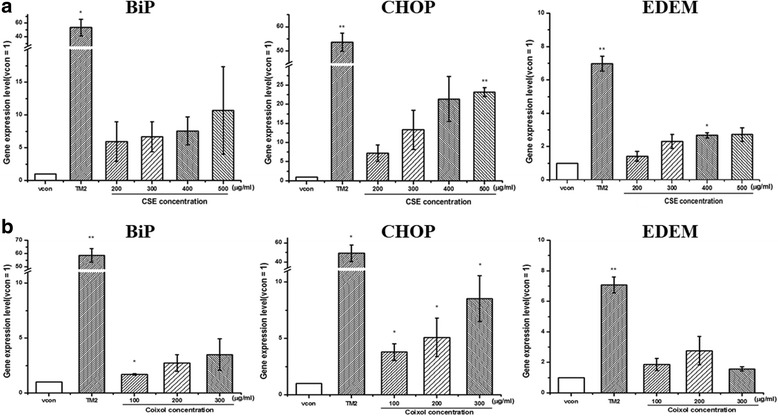
Effects of ethanol extract of Coicis Semen (CSE) and coixol on the gene expression of ER stress markers in Chang liver cells. **a** CSE 200, 300, 400, or 500 μg/mL; **b** coixol 100, 200, or 300 μg/mL. Data are presented as the mean ± SEM from three independent experiments and were compared with the vehicle control [0.5% dimethyl sulfoxide (DMSO)]. TM: tunicamycin; *: *p <* 0.05, **: *p <* 0.01. β-Actin was used as the reference gene to normalize the data using the ΔΔCt method

Expression levels of the genes for the targeted markers of ER stress–BiP, CHOP, and EDEM–were affected by different concentrations of CSE (Fig. 2a) and coixol (Fig. 2b). As shown in Fig. 2, CSE and coixol increased the gene expression of BiP and CHOP in a dose-dependent manner, although CSE had a higher effect on gene expression compared with coixol when used at the same concentration. CSE dose-dependently increased the gene expression of EDEM, whereas, increased expression of EDEM resulted by coixol did not show dose-dependent manner. These findings suggest that high concentrations of CSE or coixol can induce ER stress.

### Effects of CSE and coixol on splicing of XBP1 mRNA

**Fig. 3:**
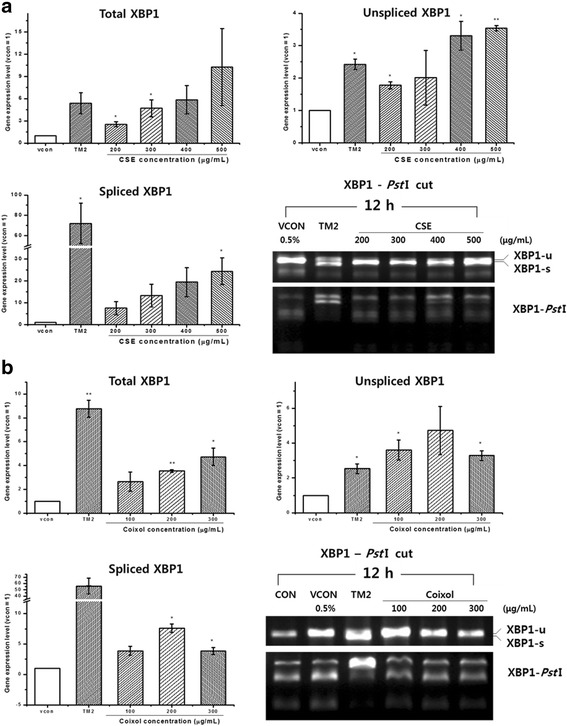
Effects of ethanol extract of Coicis Semen (CSE) and coixol on the gene expression of total XBP1, unspliced XBP1, spliced XBP1, and XBP1 cut by *Pst*I in Chang liver cells. **a** CSE 200, 300, 400, or 500 μg/mL; **b** coixol 100, 200, or 300 μg/mL. Data are presented as the mean ± SEM from three independent experiments and were compared with the vehicle control [0.5% dimethyl sulfoxide (DMSO)]. TM: tunicamycin;*: *p <* 0.05, **: *p <* 0.01. β-Actin was used as the reference gene to normalize the data using the ΔΔCt method

Using qRT-PCR, we examined the effects of CSE and coixol on the expression of total, unspliced and spliced XBP1. As shown in Fig. 3a, CSE dose-dependently increased the the expression of XBP1. The total XBP1 amplified by qRT-PCR was cut using the restriction enzyme *Pst*I and the fragments were evaluated. Expression of the fragments produced by *Pst*I cleavage was decreased by 400 and 500 μg/mL CSE. The expression of total XBP1 was increased by coixol in a dose-dependent manner. In contrast, the expression of unspliced and spliced XBP1 were not commensurate with an increasing concentration of coixol. No spliced XBP1 band (uncut by *Pst*I) was observed at coixol concentrations of 100, 200, or 300 μg/mL (Fig. 3b). Our results suggest that CSE caused ER stress by inducing the UPR through the IRE1 pathway, which activated ER chaperones and other transcription factors related to the UPR.

### Effects of different concentrations of CSE and coixol on the protein levels of ER stress markers

**Fig. 4:**
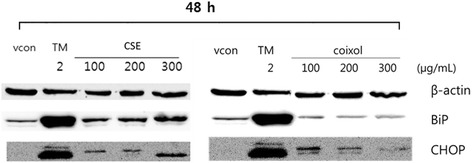
Effects of ethanol extract of Coicis Semen (CSE) and coixol on protein expression as determined by western blot analysis. Western blotting results after treatment with CSE (100, 200, or 300 μg/mL) orcoixol (100, 200, or 300 μg/mL) for 24 or 48 h in Chang liver cells. Tunicamycin (TM) was used as the positive control. Data are presented as the mean ± SEM from at least three independent experiments and were compared with the vehicle control [0.5% dimethyl sulfoxide (DMSO)]. *: *p <* 0.05, **: *p <* 0.01. β-Actin was used as the control

Western blot analysis was used to investigate the expression of ER stress marker proteins such as BiP and CHOP. Chang liver cells were treated with different doses of CSE or coixol (100, 200, or 300 μg/mL) for 48 h. Fig. 4 shows that CSE increased BiP and CHOP protein levels. The highest increase in CHOP protein level was induced by CSE at a concentration of 300 μg/mL. This suggests that a high dose of CSE caused upregulation of CHOP, which led to ER-mediated apoptosis. Even thought, coixol treatment induced only a small change in BiP protein level, 100 μg/mL coixol induced the highest level of CHOP protein level. Since coixol caused only a low level of ER stress, and the CSE-induced that most of changes in ER stress, which might possibly be caused by components of CSE other than coixol.

### Association of CSE-induced ER stress with protein-folding capacity

**Fig. 5:**
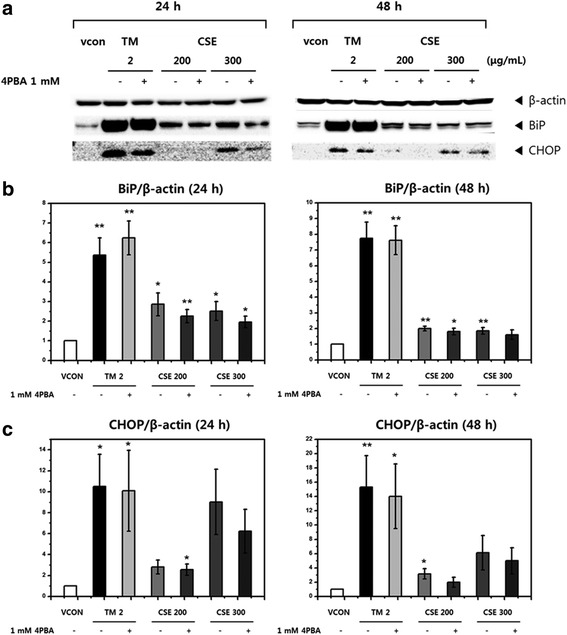
Effects of ethanol extract of Coicis Semen (CSE) and 4-phenylbutyric acid (4-PBA) on protein expression as determined by western blot analysis. **a** Western blot analysis of the levels of ER stress markers (BiP and CHOP) after treatment with CSE (200 or 300 μg/mL) for 24 h or 48 h with or without 1 mM 4-PBA in Chang liver cells, (**b**) Quantitative graph showing the levels of BiP and (**c**) CHOP. Tunicamycin (TM, 2 μg/mL) was used as the positive control, and 1 mM 4-PBA was used to inhibit ER stress. Data are presented as the mean ± SEM from at least three independent experiments and were compared with the vehicle control [0.5% dimethyl sulfoxide (DMSO)]. *: *p <* 0.05, **: *p <* 0.01. β-Actin was used as the control

We compared the effects of tunicamycin and CSE on ER stress in the presence or absence of 1 mM 4-PBA (Fig. 5a). Chang liver cells were treated with 1 mM 4-PBA and CSE for 48 h. As shown in Fig. 5b, BiP expression was not significantly affected by 4-PBA in the presence of tunicamycin-induced or CSE-induced ER stress. As shown in Fig. 5c, CHOP expression in the tunicamycin-treated or CSE-treated cells decreased after 24 h and 48 h treatments with 4-PBA, but was not significantly lower than the that of a single treatment with tunicamycin. These data suggest that 4-PBA treatment may decrease CHOP protein expression by inhibiting ER stress.

### Changes in the expression of UPR-mediated protein following CSE treatment

**Fig. 6:**
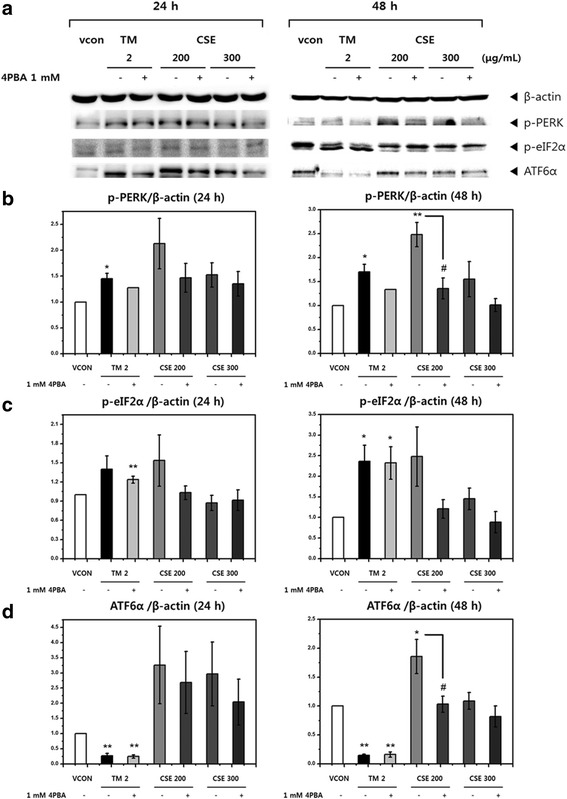
Effects of ethanol extract of Coicis Semen (CSE) on the expression of unfolded protein response (UPR)-associated proteins. **a** Western blot analysis of the levels of UPR-associated proteins (p-PERK, p-eIF2α and ATF6α) after treatment with CSE (200, or 300 μg/mL) for 24 h or 48 hwith or without 1 mM 4-PBA in Chang liver cells, (**b**) Quantitative graph of the levels of p-PERK, (**c**) p-eIF2α and (**d**) ATF6α. Tunicamycin (TM, 2 μg/mL) was used as the positive control and 1 mM 4-PBA was used to inhibit ER stress. Data are presented as the mean ± SEM from at least three independent experiments and were compared with the vehicle control [0.5% dimethyl sulfoxide (DMSO)]. *: *p <* 0.05, **: *p <* 0.01, #: Significant difference (*p* < 0.05) compared with treatment with CSE alone (200 μg/mL).β-Actin was used as the control

Tunicamycin and CSE increased the protein levels of p-PERK and p-eIF2α. Similarly, CSE appeared to increase the protein level of glycosylated ATF6α (90 kDa), although the data were not conclusive. To confirm whether 4-PBA can inhibit ER stress via the PERK and ATF6α pathways, we examined the effects of 4-PBA on p-PERK, p-eIF2α, and ATF6α protein levels. There were non-significant decreasing tendencies in the amount of p-PERK and p-eIF2α in cells exposed to 4-PBA (Fig. 6a), with the exception of the change in expression of p-PERK when cells were exposed to 200 μg/mL CSE for 48 h both with and without 1 mM 4-PBA. Figure 6b and c show that ER stress induced by CSE or tunicamycin could be reduced by 4-PBA treatment. This suggests that tunicamycin and CSE can increase activation of the PERK pathway, and 4-PBA might prevent the accumulation of unfolded proteins by improving folding capacity in the ER.

Figure 6a shows a protein expression of deglycosylated ATF6α that appeared below the glycosylated ATF6α (90 kDa) after treatment with tunicamycin. These results suggest that tunicamycin induced ER stress and stimulated the activation of the ATF6α pathway to trigger deglycosylation. Treatment with CSE (200, or 300 μg/mL**)** increased the level of ATF6α (90 kDa). 4-PBA significantly decreased the amount of ATF6α when administered with 200 μg/mL CSE for 48 h (Fig. 6d). However, no significant decrease in ATF6α (90 kDa) was observed following treatment with other concentrations of CSE.

## Discussion

Adverse effects related to the indiscriminate use of medicinal plants noted recently highlight the need for toxicological evaluation of these plants. This study was conducted to investigate the toxicity and ER-stress caused by CSE, and its related protein and gene expressions.

In previous studies, Coicis Semen has been shown to inhibit the proliferation of HepG2 [18], Calu-6, MCF-7, and SNU-601 cells [19]. Cong-yan L et al., [20] reported IC_50_ values of 1.64 and 2.33 mg/mL for CSE (from Hebei, China) against human lung cancer SPC-A-1 and A549 cells, respectively. On the other hand, a methanol extract of Coicis Semen induced slight apoptosis in HepG2 cells when used above a concentration of 1000 μg/mL [21]. Although many studies have reported on the cytotoxicity of Coicis Semen against cancer cells, we cannot be sure that Coicis Semen affects the proliferation of cancer cells without also affecting normal cells. Our data suggest that high concentrations of Coicis Semen (IC_50_; 250 μg/mL) and coixol (IC_50_; 350 μg/mL) can induce damage in normal liver cells.

When exposed to stress, the ER upregulates BiP gene expression to increase protein-folding capacity, and promotes the ERAD pathway to increase the expression of ERAD-associated components such as EDEM. If irreversibly damaged, the ER induces apoptosis by increasing the synthesis of CHOP via the UPR pathway [22]. CHOP is the most inducible gene during strong ER stress and a key indicator of ER-mediated apoptosis [23]. Generally, CHOP expression is very low in the normal state but its expression increases markedly in response to severe ER damage. In this study, the gene expression of ER stress markers such as BiP, CHOP, and EDEM was increased by treatment with CSE or coixol. The toxic effect of CSE on ER stress was greater than that of coixol when used at the same concentration, as well as the toxicity might also depend on their constituents in the herbal mixture.

Our study focused specifically on deleterious effects of ER-stress on the alternative systems which induced by UPR in abnormal cells. In the process, changes in the ER-mediated proteins were reflected by changes in the three sensor proteins (IRE1, PERK, and ATF6α). XBP1 mRNA is spliced by activated IRE1 in the ER membrane. Yoshida et al., [24] reported that indicating the splicing of XBP1 mRNA upon ER stress is a good marker of ER stress. PERK membrane protein initiates adaptive responses by oligomerization and trans-autophosphorylation [25], whereas p-PERK catalyzes the phosphorylation of eIF2α, which attenuates global protein translation during the UPR [26, 27]. The expression level of ATF6α (90 kDa) decreased after treatment of HeLa cells with 2 μg/mL tunicamycin for 4 h [28]. Hong et al., [29] reported that tunicamycin stimulated deglycosylation of ATF6α and that the deglycosylated form exhibited a faster rate of constitutive transport to the Golgi. Li et al., [30] reported that the synthetic steroidal glycoside SBF-1 decreased the expression level of ATF6 (90 kDa), but increased that of ATF6α (50 kDa).

In the present study, the protein expression of spliced XBP1 was observed after exposure to high-dose CSE (≥400 μg/mL), and the gene expression of spliced XBP1 increased in a dose-dependent manner. We examined the effect of tunicamycin treatment in the presence or absence of 4-PBA to compare its effect with that of CSE on the ability to upregulate the expression of ER-mediated proteins. Treatment of human hepatic cells with CSE also caused changes in ER stress markers. Expressions of BiP, CHOP, EDEM, and sXBP1 increased with an increasing concentration of CSE, although the CSE-induced increase in expression of p-PERK and p-eIF2α was not significant. These increased expressions of CHOP, p-PERK, and p-eIF2α induced by CSE were decreased by treatment with 4-PBA. Similarly, tunicamycin (1 μg/mL), which induced stacking of unfolded protein in the ER membrane by inhibiting N-linked glycosylation, increased the levels of p-PERK, p-eIF2α, CHOP, ATF6, and BiP proteins.

Treatment with 4-PBA (5 mM), which inhibited ER stress, attenuated the tunicamycin-induced increase in ER stress-related protein expressions in endothelial cells [31]. In contrast, BiP expression was not significantly affected by tunicamycin-induced ER stress in the presence of 4-PBA, which was similar result with Malo et al., [32] reported of preincubation with 4-PBA significantly decreased BiP expression. The expression of full ATF6α (90 kDa glycosylated form) was also increased by CSE. Similarly, 100 μM of 6-hydroxydopamine led to an increase in the expression of ATF6α mRNA and protein in PC12 cells [33]. However, it is not sufficient to fully understand direct association between ER stress induced by CSE and the increment of ATF6α caused by CSE, because we did not observe the fragmentation of ATF6α (50 kDa). Thus, we suggest that the mechanism responsible for CSE-induced ER stress is mediated via the PERK and IRE1 pathways. However, at present, it is difficult to pinpoint the active constituents that cause the observed ER stress induced by CSE. Further studies are needed to identify the chemical constituents of CSE that cause ER stress in human liver cells.

## Conclusion

In conclusion, a high dose of CSE (≥200 μg/mL) or coixol was cytotoxic to Chang liver cells, which suggests that CSE can induce ER stress in human liver cells. Thus, the CSE and coixol may induce ER stress and stimulate the UPR via activation of the PERK and IRE1 pathways in normal liver cells.

## Additional file

Additional file 1: **Figure S1.** Chromatographic profile of the ethanol extract of Coicis Semen (CSE). The inserted figure shows the standard curve for the coixol concentration versus area. (TIFF 78 kb)

## Abbreviations

4-PBA: Sodium 4-phenylbutyrate
ATF6α: Activating transcription factor 6 alpha
BiP: Binding immunoglobulin protein
CHOP: CCAAT/enhancer binding protein homologous protein
CSE: Coicis semen extract
DMSO: Dimethyl sulfoxide
eIF2α: Eukaryotic translation initiation factor 2 alpha
ER: Endoplasmic reticulum
ERAD: ER-associated degradation
FBS: Fetal bovine serum
HPLC: High-performance liquid chromatography
HRP: Horseradish peroxidase
IRE1: Inositol-requiring protein 1
PERK: Protein kinase-like endoplasmic reticulum kinase
qRT-PCR: Quantitative reverse transcription polymerase chain reaction
RIPA: Radioimmunoprecipitation assay
UPR: Unfolded protein response
XBP1: X-box binding protein 1
