# Supplementary figures and images for: Endoplasmic reticulum stress induced by an ethanol extract of Coicis semen in Chang liver cells
Source: BMC Complement Altern Med. 2018 Mar 20;18:100. doi: 10.1186/s12906-018-2175-z (PMC5859727; doi:10.1186/s12906-018-2175-z)

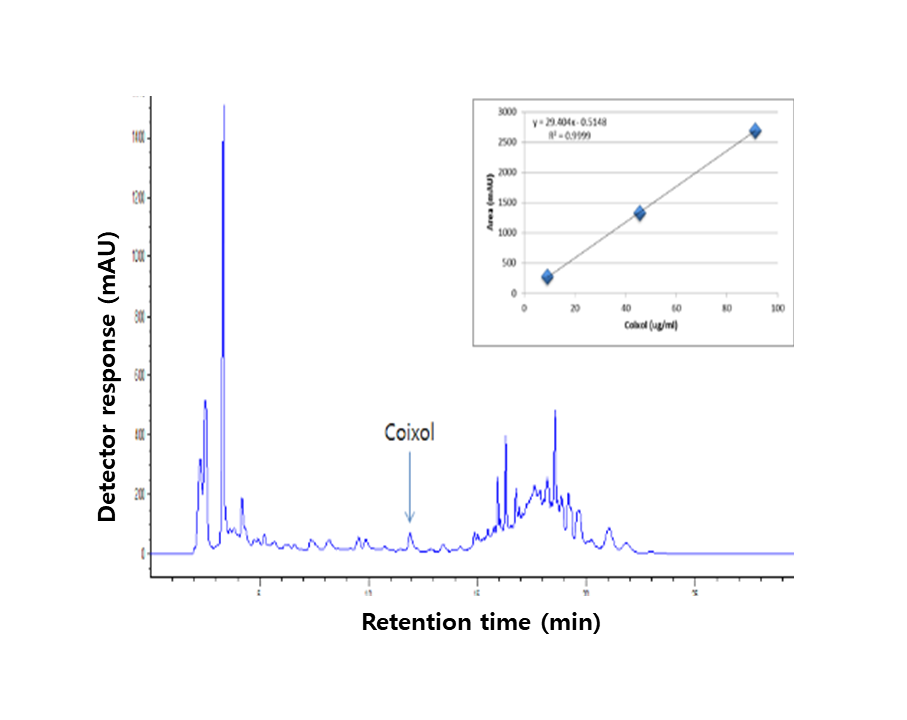

Supplement: Supplementary file 1 — Figure S1. Chromatographic profile of the ethanol extract of Coicis Semen (CSE). The inserted figure shows the standard curve for the coixol concentration versus area. (TIFF 78 kb) [file 12906_2018_2175_MOESM1_ESM.tif]
